# Advancing Cardiovascular Risk Stratification and Functional Assessment: A Narrative Review of CPET and ESE Applications

**DOI:** 10.3390/healthcare13131627

**Published:** 2025-07-07

**Authors:** Valerio Di Fiore, Lavinia Del Punta, Nicolò De Biase, Stefano Masi, Stefano Taddei, Javier Rosada, Michele Emdin, Claudio Passino, Iacopo Fabiani, Nicola Riccardo Pugliese

**Affiliations:** 1 Department of Clinical and Experimental Medicine, University of Pisa, Via Roma 67, 56124 Pisa, Italy; difiore95@hotmail.it (V.D.F.); laviniadelpunta@gmail.com (L.D.P.); nicolo.debiase94@gmail.com (N.D.B.); stefano.masi@unipi.it (S.M.); stefano.taddei@unipi.it (S.T.); n.r.pugliese88@gmail.com (N.R.P.); 2 Fourth Unit of Internal Medicine, University Hospital of Pisa, 56124 Pisa, Italy; javier_rosada@hotmail.it; 3 Division of Cardiology and Cardiovascular Medicine, Fondazione Toscana Gabriele Monasterio—FTGM, 56122 Pisa, Italy; m.emdin@santannapisa.it (M.E.); claudio.passino@santannapisa.it (C.P.); 4 Scuola Superiore Sant’Anna—Sant’Anna School of Advanced Studies, 56127 Pisa, Italy

**Keywords:** cardiopulmonary exercise testing, exercise stress echocardiography, exercise hemodynamics

## Abstract

Cardiopulmonary exercise testing combined with exercise stress Echocardiography (CPET-ESE) is an advanced diagnostic modality for evaluating cardiovascular disease and tailoring patient-specific treatment strategies. By integrating metabolic, ventilatory, and hemodynamic data with real-time imaging, CPET-ESE offers a comprehensive assessment of cardiovascular function under physiological stress. CPET provides detailed insights into metabolic and ventilatory performance, while ESE allows for the dynamic visualisation of cardiac structure and function during exercise. This review outlines the physiological foundations and core parameters of CPET and ESE, emphasising their complementary roles in cardiovascular diagnostics and prognostication and exploring their clinical value for evaluating unexplained dyspnoea and exercise-induced hemodynamic abnormalities. CPET-ESE plays a pivotal role in detecting subtle hemodynamic abnormalities, assessing functional capacity, and contributing to earlier diagnosis, targeted interventions, and improved clinical outcomes.

## 1. Introduction

Exercise intolerance results from an impaired interplay between the cardiovascular, pulmonary, and muscular systems during physical activity. Particularly, it can result from cardiac dysfunction, lung disease, or reduced peripheral oxygen extraction due to impaired muscle metabolism [1].

Cardiopulmonary exercise testing (CPET) has emerged as a pivotal tool in evaluating exercise intolerance in various pathological conditions. Although CPET allows for a detailed analysis of the physiological or pathological response to exercise, it does not distinguish its central (i.e., ventricular function) and peripheral components (i.e., oxygen delivery and extraction) unless exercise is carried out together with a concurrent invasive hemodynamic evaluation by cardiac catheterization [2] or with imaging techniques such as stress echocardiography (ESE) [1,3]. As a non-invasive method, ESE has been successfully used to gain insight into cardiovascular mechanisms underpinning effort intolerance in patients with different cardiovascular diseases [4,5].

The combined approach with CPET-ESE provides complementary yet distinct sets of parameters (Graphical Abstract) for analysing metabolic and ventilatory responses during exercise, enabling a more direct assessment of cardiovascular performance [1]. Moreover, while the earliest alterations of cardiovascular diseases become evident during physical effort, this technique can timely reveal pathologies in the initial stages before the onset of overt symptoms. All these features make this test a powerful tool to optimise decision-making, improve outcome prediction, and provide objective values for tailored treatment also in the early stages of several diseases such as heart failure (HF), cardiomyopathies, valvulopathies, coronary artery disease (CAD), pulmonary hypertension (PH), and pulmonary embolism (PE) sequelae [6]. Moreover, it represents a tool for risk stratification in heart and lung transplantation and pre-surgical evaluations [7,8,9,10,11,12,13]. Currently, a standardised protocol for combined CPET-ESE has not been established. However, adhering to general recommendations can enhance the reliability and consistency of the results [12]. This narrative review explores the clinical value and integration of the CPET-ESE protocol for evaluating unexplained dyspnoea and exercise-induced hemodynamic abnormalities.

## 2. Methods

This narrative review was conducted to summarise and interpret the existing literature on the integration of CPET and ESE for clinical evaluation and risk stratification, with the aim to answer a simple question: how can CPET-ESE contribute to improving the understanding and detection of exercise-induced hemodynamic abnormalities in cardiovascular diseases? To ensure a comprehensive and up-to-date review of the literature, we conducted a structured search of relevant scientific databases. Specifically, the databases PubMed and Scopus were searched for articles related to CPET, ESE, and their combined application in the evaluation of HF, cardiomyopathies, valvulopathies, CAD, and pulmonary diseases.

The search included publications from 1991 to 2025. Keywords and MeSH terms used in various combinations included “cardiopulmonary exercise testing”, “CPET”, “exercise stress echocardiography”, “ESE”, “heart failure with preserved ejection fraction”, “heart failure with reduced ejection fraction”, “HFpEF”, “HFrEF”, “pulmonary hypertension”, “valvular heart disease”, “mitral regurgitation”, “mitral stenosis”, “aortic regurgitation”, “aortic stenosis”, “hypertrophic cardiomyopathy”, “prognosis”, and “diagnostic accuracy”.

The analysis included original studies, reviews, and guidelines written in English, focusing specifically on adult populations. Eligible articles involved the use of CPET and/or ESE in clinical practice or research settings and provided data relevant to diagnosis, prognosis, or therapeutic decision-making. Studies were excluded if they involved paediatric populations, lacked sufficient methodological detail, or had unclear endpoints.

## 3. Physiology and Key Parameters of CPET

The use of oxygen as the primary energy resource by peripheral tissues—i.e., oxygen consumption (VO_2_)—depends on cardiac output (CO), on the efficient distribution of the substrate to active tissues, and finally on their ability to extract the substrate from the bloodstream. This is represented in Fick’s principle, whereby VO_2_ is calculated as the product between CO (i.e., heart rate [HR] x stroke volume [HR]) and the arteriovenous oxygen difference (AVO_2_diff), which is the difference between arterial and venous oxygen content, reflecting how much oxygen is extracted by the muscles during exercise [14].

VO_2_ increases up to six-fold at peak exercise in healthy adults, indicating good functional capacity and prognosis. Lower values, particularly in conditions like HF, are associated with poorer outcomes [7,15]. The VO_2_/work ratio reflects the efficiency of VO_2_ relative to the external workload and can help identify physical deconditioning or left ventricular dysfunction, particularly when myocardial ischemia is present.

The other crucial component of effort capacity is ventilatory efficiency. The minute ventilation–carbon dioxide production (VE/VCO_2_) slope is an index of ventilation–perfusion matching in the lung and describes the increase in minute ventilation for any given amount of CO_2_ generated from cellular respiration [16,17]. It is determined by CO_2_ production, the physiological dead space to tidal volume ratio (VD/VT), and the arterial CO_2_ partial pressure. End-tidal CO_2_ pressure (PETCO_2_) represents the partial pressure of CO_2_ in exhaled air; thus, it is an indirect marker of alveolar ventilation and ventilation–perfusion matching [18]. The breathing reserve (BR), resulting from the difference between maximal voluntary ventilation (MVV) and VE, indicates the ventilatory capacity during exertion [17,19]. Exercise Oscillatory Ventilation (EOV) is an ominous sign that refers to cyclic fluctuations in ventilation and expired gas exchange during exercise, commonly observed in individuals with advanced diseases and poor prognosis (Table 1).

## 4. Physiology and Key Parameters of ESE

ESE allows for the evaluation of chamber geometry and volumes, left ventricle (LV) and right ventricle (RV) function, and valvular responses to exercise (Table 2).

The inability to increase the LVEF ≥ 7.5% from rest to peak exercise identifies the absence of contractile reserve, which can be related to the progressive impairment of the cardiac mechanics and/or the coronary flow reserve. Other parameters have been proposed as earlier markers of LV contractility, namely Tissue Doppler-derived early systolic velocity of the mitral annulus (TDI-S’) and Speckle tracking-derived global longitudinal strain (GLS) [7].

The diastolic function can be thoroughly assessed by utilising various techniques that provide insights into the diastolic response to exercise. Peak Tricuspid Regurgitation Velocity (TRV) evaluates the degree of regurgitant flow through the tricuspid valve. Systolic pulmonary arterial pressure (sPAP) is estimated during RV systole to assess pulmonary hemodynamics [29].

The average E/e’ ratio, which is the ratio of transmitral flow velocity to mitral annular velocity in early diastole, correlates well with left ventricular filling pressure when assessed at rest [36]. Still, doubts have been recently raised regarding the technical feasibility and reliability of this parameter at rest [52,53] and during exercise [37].

The assessment of heart valve disease relies on various echocardiographic parameters that measure the severity of regurgitation and stenosis by evaluating flow dynamics, orifice size, and pressure gradients. The vena contracta is the narrowest part of the regurgitant jet and provides a semi-quantitative estimate of its severity; its value can vary with physical exertion and is influenced by technical and flow-related factors [50]. The effective regurgitant orifice area (EROA) quantitatively measures the effective regurgitant orifice area, offering a direct indication of severity and is also affected by hemodynamic conditions and the type of regurgitation [50]. Regarding valvular stenosis, peak velocity and mean pressure gradient (MPG) measured by Doppler allow for the assessment of the degree of narrowing; both are flow-dependent and can be influenced by factors such as cardiac output, blood pressure, and vascular compliance [18,29,51]. Tricuspid annular plane systolic excursion (TAPSE), a key indicator of RV systolic performance, is based on tricuspid annular displacement during systole [11]. The TAPSE/sPAP ratio is measured to assess RV–pulmonary artery (RV-PA) coupling, representing the relationship between RV function and pulmonary vascular load [45]. The left atrium (LA) is another component of the RV and RV-PA coupling evaluation, and its dysfunction contributes to reduced pulmonary vessel compliance and abnormal right heart adaptation to exercise, as indicated by its correlation with the sPAP/TAPSE ratio [35,41]. Left atrial reservoir function (LARS) quantifies LA deformation during atrial filling [39]. Similarly, the mean pulmonary artery pressure (mPAP)/CO slope is a hemodynamic parameter that reflects the coupling between pulmonary arterial pressure and CO during exercise. It considers changes in both mPAP and CO from rest to exertion [9,47,48]. Any significant valvular abnormality identified through colour Doppler imaging should be further evaluated using semi-quantitative or quantitative methods, following current guidelines, both at rest and during peak exercise [54]. Lung ultrasound (LUS) plays an essential role in assessing pulmonary congestion, particularly in the setting of HF [13,55], and it has been recently integrated into exercise testing [1]. During exercise, the increase in B-lines, rather than their absolute number at a given moment, seems a better indicator of extravascular lung water accumulation [7].

Moreover, the assessment of congestion has been enhanced by extending the evaluation of the inferior vena cava (IVC) from rest to peak exercise. While IVC measurements were already part of the baseline evaluation, they are now systematically acquired at peak effort as well. This allows for the estimation of right atrial pressure (RAP) during stress, based on dynamic changes in IVC diameter and collapsibility. Specifically, IVC metrics obtained at peak exercise are integrated to estimate exercise-induced RAP. When a significant discrepancy between resting and peak is observed, a correction can be added to the tricuspid regurgitation (TR) pressure gradient.

## 5. Integrated CPET-ESE Protocol

CPET provides a global assessment of the integrative responses involving the pulmonary, cardiovascular, and muscular systems during exercise. It is especially valuable when evaluating functional capacity and uncovering hidden mechanisms of dyspnoea. On the other hand, ESE enables the dynamic visualisation of cardiac structure and function during effort. Individually, both techniques offer useful but incomplete information. CPET lacks anatomical and hemodynamic detail, while ESE may miss functional and ventilatory parameters. Combining them into a unified protocol—CPET-ESE—offers a unique opportunity to integrate gas exchange data with simultaneous echocardiographic assessment under physiological stress.

**Figure 1:**
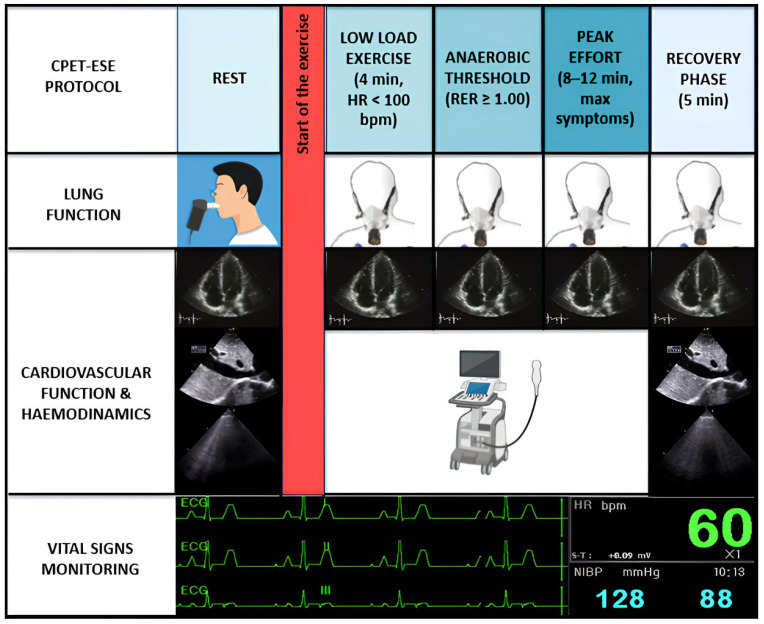
Combined CPET-ESE protocol. CPET-ESE enables an objective assessment of exercise intolerance using a graded ramp protocol to ensure gradual workload increase. Briefly, we designed a structured four-stage protocol: (i) rest, (ii) low-load effort (HR < 100 bpm), (iii) anaerobic threshold (RER ≥ 1.00), and (iv) peak effort, followed by a recovery phase. The test includes continuous breath-by-breath gas exchange analysis, real-time echocardiographic imaging, ECG, BP, and HR monitoring. Preceding spirometry helps identify pulmonary abnormalities contributing to exercise limitation.

CPET-ESE offers an objective evaluation of exercise intolerance, usually measuring each parameter according to a four-stage protocol (Figure 1): (i) rest; (ii) low-load effort (typically within the first 4 min when heart rate (HR) is usually <100 bpm); (iii) anaerobic threshold (AT, i.e., when the respiratory exchange ratio (RER) [expressed by the carbon dioxide production (VCO_2_)/VO_2_ ratio] is steady ≥1.00); and (iv) peak effort, when the patient experiences effort-limiting symptoms, within 8–12 min [1,3]. A recovery phase of approximately five minutes follows the peak exercise stage. Throughout the exercise protocol a cardiac ultrasound probe is actively used to acquire real-time echocardiographic images.

After a brief warm-up phase, a symptom-limited graded ramp test is initiated, featuring continuous breath-by-breath gas exchange analysis, along with the ongoing monitoring of blood pressure (BP) and HR. Throughout the exercise, a 12-lead electrocardiogram is performed. Likewise, a functional pulmonary evaluation using spirometry should precede CPET-ESE to identify lung abnormalities associated with exercise intolerance, particularly moderate or greater airflow obstruction (i.e., a forced expiratory volume in the first second [FEV1] to forced vital capacity [FVC] ratio < 50% of predicted), restrictive patterns (FVC < 80% of predicted), or exercise-induced bronchospasm. Incremental ramps allow for a gradual and low-intensity workload increase (8–15 W/min), different from the more abrupt increment used in the Bruce protocol stress test to diagnose CAD [7]. To collect clear echocardiographic images, patients are typically asked to exercise in a semi-reclined position, which slightly differs from standard CPET protocols using upright cycle ergometers or treadmills. However, there appears to be little to no difference in peak VO_2_ achieved between these methods. Despite its advantages, CPET-ESE remains underutilised due to logistical challenges and the need for trained multidisciplinary teams. Further studies are warranted to validate its prognostic value and integrate it into routine clinical algorithms.

## 6. CPET-ESE Role in the Diagnosis and Risk Stratification of Cardiovascular Diseases

### 6.1. HF Spectrum

CPET-ESE may hold a pivotal tool in the diagnosis and risk stratification of patients with HFrEF (LVEF < 40%), preserved ejection fraction (HFpEF, LVEF ≥ 50%), or mid-range LVEF (40–49%, HFmrEF).

Patients with HF often suffer from multiple comorbidities, making it difficult to rely solely on clinical data; thus, CPET-ESE helps clinicians to identify the pathophysiologic and prognostic impact of cardiovascular and ventilatory alterations contributing to exercise intolerance [7,56]. Moreover, this technique allows for the early identification of patients with initial impairment before symptoms manifest at rest. The transition from cardiometabolic conditions, such as hypertension and diabetes, to overt HF is often subtle, and in the early stages, it only becomes evident under physiological stress [21,57,58,59]. Reduced peak VO_2_ values, associated with decreased AVO_2_diff and mild signs of left ventricular systolic dysfunction, have been shown in hypertensive, asymptomatic subjects. Similarly, pulmonary congestion markers, namely a steeper VE/VCO_2_ slope and increased B-lines, have been observed in hypertensive patients and latent HFpEF [8]. Increased central arterial stiffness and altered BP pulsatility significantly correlate with reduced peak VO_2_ in a similar population of hypertensive subjects [17]. Similarly, in the evaluation of ventricular–arterial coupling through resting, Doppler-derived proximal aortic stiffness (aa-PWV) has proven feasible and reproducible across the heart failure spectrum, with the aa-PWV/GLS ratio emerging as an independent predictor of peak VO_2_ and functional capacity impairment [60]. Hypertensive response to exercise is also associated with impaired functional capacity across the HF spectrum [21].

In HFmrEF patients, the reduced peak VO_2_ observed during CPET may be primarily driven by impaired AVO_2_diff, suggesting a physiological behaviour closer to HFpEF than HFrEF. This diminished peripheral oxygen extraction capability—commonly observed in “poor extractors”—highlights the potential value of interventions aimed at enhancing skeletal muscle perfusion and oxygen utilisation in this subgroup. Combined CPET-ESE thus enhances phenotypic characterisation and functional assessment in HFmrEF, bridging the diagnostic and therapeutic gap between reduced and preserved ejection fraction phenotypes [61].

Pulmonary comorbidities play a crucial role in the progression and symptom burden of HF, especially in HFpEF, as follows: increased pulmonary capillary wedge pressure during exertion leads to fluid accumulation in the lungs, triggering vascular dysfunction and impaired pulmonary mechanics. This results in ventilation inefficiency and exercise-induced dyspnoea, which are common in HFpEF patients. In patients with overt or suspected HFpEF, CPET-ESE helps investigate the relationship between exercise-induced pulmonary hypertension, excess lung water accumulation, and ventilation–perfusion mismatch [8,17]. The detection of an exaggerated rise in pulmonary vascular resistance during exercise, impaired pulmonary diffusing capacity, and increased VD/VT indicate a maladaptive pulmonary hemodynamic response. Such findings correlate with disease progression and poorer prognosis, highlighting the critical role of pulmonary dysfunction in limiting functional capacity and worsening symptoms in HFpEF patients [62,63,64].

### 6.2. Aortic Stenosis and Regurgitation

Severe symptomatic aortic stenosis (AS) is typically an indication of aortic valve replacement. However, many patients with degenerative aortic stenosis (AS)—especially elderly individuals in Western countries—may appear asymptomatic due to activity self-limitation or may report non-specific dyspnoea stemming from a variety of extra-valvular and extracardiac comorbidities [54]. In such cases, CPET-ESE proves highly valuable by objectively quantifying exercise intolerance via peak VO_2_ and correlating findings with additional CPET-ESE parameters to determine dyspnoea aetiology. Furthermore, CPET-ESE helps detect the worsening of AS during exertion, impaired functional capacity, and the onset of symptoms, which can guide changes in the patient’s therapeutic approach [29]. ESE examination is critical as it evaluates the hemodynamic severity of the condition. This includes assessing peak transvalvular velocity, mean transvalvular pressure gradient, estimated aortic valve area, and the presence or absence of LV functional flow or contractile reserve (i.e., appropriate SV or LVEF increase during exercise). ESE can also identify dynamic increases in sPAP and pulmonary congestion throughout exercise, which carry negative prognostic implications [65]. Meanwhile, CPET correlates echocardiographic signs of pulmonary congestion with data on ventilation–perfusion mismatch, specifically through the VE/VCO_2_ slope. These combined measures significantly enhance prognostic assessment and risk stratification in AS patients [1]. Recently, mPAP/CO, a sign of pulmonary hypertension, has been proposed in AS evaluation as an early marker of pulmonary vascular and myocardial maladaptation. A high mPAP/CO slope and low peak VO_2_ resulted in worse outcomes, including HF hospitalisation, atrial fibrillation, and cardiovascular mortality [9]. Their integration into clinical evaluations could refine decision-making regarding aortic valve replacement and provide insights into disease progression beyond conventional severity markers such as aortic valve area and LA volume index.

Similarly, CPET-ESE plays a crucial role in assessing aortic regurgitation (AR), mainly in asymptomatic patients, allowing for the detection of early signs of LV decompensation, such as an insufficient increase in LVEF, impaired contractile reserve, or even subclinical systolic dysfunction with GLS. Additionally, ESE can evaluate exercise-induced aortic regurgitation and identify patients at risk of deterioration, aiding in optimal treatment planning and follow-up [66]. An increase in sPAP or the development of exercise-induced mitral regurgitation are indicators of progression and may necessitate early intervention [66,67,68]. In chronic AR, the LV faces a significant volume overload, which, if prolonged, results in left ventricular dilation and secondary increases in LA pressures. Elevated LV pressures are transmitted backwards into the pulmonary circulation, predisposing those affected to the development of secondary PH. Under exercise conditions, the mismatch between increased cardiac output and fixed pulmonary vascular abnormalities is expected to steepen the mPAP/CO slope. Chronic pressure overload from PH may also progressively impair RV function, lowering the TAPSE/sPAP ratio.

### 6.3. Mitral Regurgitation and Stenosis

In patients with asymptomatic or mildly symptomatic mitral regurgitation (MR), CPET-ESE is a valuable tool to assess functional capacity and unmask the underlying causes of any limitations. MR severity assessment at rest and peak exercise includes semi-quantitative techniques (vena contracta) and quantitative indices (effective regurgitant orifice area, regurgitant volume, and regurgitant fraction). By tracking these parameters throughout exercise, CPET-ESE unveils the progression of valvular dysfunction and its impact on functional performance [29]. ESE is particularly useful when there is a discrepancy between the severity of MR observed at rest and the presence of symptoms, as it can reveal an abnormal hemodynamic response that suggests the need for closer monitoring or early surgical intervention. A rise in LV filling pressure can be observed, subsequently affecting the pulmonary circulation, leading to increased sPAP, lung congestion, and ventilation–perfusion mismatch. An increase in sPAP beyond 60 mmHg during ESE has been linked to symptom onset, offering a valuable diagnostic tool for identifying patients at higher risk of deterioration despite appearing asymptomatic at baseline [69]. These signs are reflected in a steeper VE/VCO_2_ slope. In more severe cases, EOV may occur, particularly in patients with secondary MR, such as those with tethered mitral valve leaflets in a dilated and dysfunctional LV [29]. CPET-derived parameters, particularly reduced peak VO_2_, have been associated with increased mortality and HF hospitalisations. Moreover, the inability to achieve an adequate exercise response may indicate a higher risk of major adverse cardiovascular events. By identifying patients with poor exercise tolerance and impaired cardiopulmonary reserve, CPET-ESE helps stratify risk and optimise therapeutic strategies, reinforcing its role as a key prognostic tool in MR evaluation [70]. In MR, the regurgitant volume directly increases LA pressures even at rest, leading to chronic pulmonary venous hypertension. Exercise exacerbates this hemodynamic burden by increasing the regurgitant fraction and further elevating pulmonary artery pressures. Consequently, the limited ability of pulmonary circulation to accommodate the increased flow results in an abnormal mPAP/CO slope during exertion [71]. Simultaneously, the sustained pressure overload and remodelling of the pulmonary vasculature eventually impair RV performance, contributing to a decreased TAPSE/sPAP ratio, especially during dynamic conditions like exercise. It has been demonstrated that the mPAP/CO slope and TAPSE/sPAP slope correlate with simultaneously measured invasive catheterisation values and accurately predicted effort intolerance, independently of exercise-induced changes in MR severity or peak pulmonary artery pressures [47].

ESE is an essential tool in evaluating mitral stenosis (MS), particularly in cases where resting echocardiography may underestimate its severity. The exercise can reveal significant increases in mean transvalvular gradient and sPAP, indicating hemodynamic impairment. A marked rise in mPAP or sPAP during exertion suggests limited hemodynamic reserve and a higher likelihood of symptom progression. These findings support decision-making regarding balloon valvuloplasty or surgical intervention. ESE becomes particularly valuable in patients preparing for major surgery or pregnancy, as it predicts the likelihood of decompensation with increased cardiac demand [66]. MS creates a fixed obstruction to left ventricular filling, causing the persistent elevation of LA pressure, which is directly transmitted to the pulmonary veins and arteries. As a result, secondary PH is a common complication, often worsening under stress or exercise. The inability of the pulmonary vasculature to handle increased CO during exertion leads to a disproportionate rise in pulmonary artery pressures, manifesting as an increased mPAP/CO slope. Prolonged PH in MS also exposes the RV to chronic afterload stress, progressively leading to RV-PA uncoupling and a reduced TAPSE/sPAP ratio.

### 6.4. Hypertrophic Cardiomyopathy

Hypertrophic cardiomyopathy (HCM) is a multifaceted condition, with several factors that could contribute to exercise limitation, including left ventricular diastolic dysfunction, left ventricular outflow tract (LVOT) obstruction, and potential peripheral muscle alterations. Around 5% of patients with HCM develop systolic dysfunction, with ventricular wall thinning and cavity dilation, leading to exercise intolerance mechanisms similar to those seen in systolic HF. A key determinant of reduced exercise capacity in HCM is the inability to increase SV [72]. Exercise capacity seems primarily influenced by reduced SV, which correlates inversely with the time to peak left ventricular filling [73]. Further studies have confirmed that peak VO_2_ is closely related to peak cardiac index, itself linked to diastolic function markers such as E/e’ [74]. LVOT obstruction significantly limits SV augmentation during exercise and can exacerbate mitral regurgitation, elevate pulmonary pressures, and further impair diastolic function by prolonging systole. ESE can reveal stress-induced LVOT obstruction, worsening mitral regurgitation, and latent diastolic dysfunction. Chronotropic incompetence is another factor that contributes to exercise limitation in HCM. While its exact mechanisms are not fully understood, potential causes include sinoatrial node remodelling, altered beta-receptor function, and impaired intracellular calcium signalling [75]. Finally, many HCM patients experience deconditioning due to reduced activity levels, which can impair peripheral oxygen extraction [76]. Current HCM Guidelines suggest CPET for identifying candidates for septal reduction procedures [77]. Moreover, an independent relationship between peak VO_2_, VE/VCO_2_ slope, and HF-related outcomes (i.e., death and transplantation [78]) has been demonstrated.

Diastolic dysfunction is a hallmark feature in HCM, resulting from impaired ventricular relaxation and increased myocardial stiffness. This leads to elevated LV end-diastolic pressure and subsequent elevation of left atrial pressure. Over time, the chronic rise in pulmonary venous pressure can induce post-capillary PH. During exercise, the inability of the stiff LV to accommodate increased preload exacerbates pulmonary pressures, leading to a steeper mPAP/CO slope. Furthermore, the chronic RV afterload stress may impair RV-PA coupling, manifesting as a reduced TAPSE/sPAP ratio. It has been demonstrated that impaired TAPSE and a reduced TAPSE/sPAP ratio during exercise are independent predictors of adverse outcomes in patients with non-obstructive HCM [79].

### 6.5. CAD

CPET-ESE is particularly useful in evaluating patients with CAD when traditional stress testing is inconclusive, as it can detect abnormalities in oxygen delivery and utilisation before overt ischemia occurs [10]. Moreover, ESE is a valuable tool for detecting CAD by assessing regional wall motion abnormalities induced by ischemia, although CPET-ESE is less sensitive than the Bruce protocol in diagnosing CAD [80]. ESE’s high specificity makes it particularly effective in distinguishing true ischemic abnormalities from non-obstructive causes, especially in women, where microvascular dysfunction and endothelial abnormalities may contribute to ischemic symptoms despite normal coronary angiography [81]. HR response is a CPET parameter evaluated in real-time per the Fick equation. The ΔHR–WR slope (change in heart rate to work-rate slope) compares the slope of heart rate increase during the final 2 min of exercise to that during the mid-exercise phase, providing insight into cardiovascular response and potential chronotropic incompetence. Values >15% (positive ΔHR–WR slope) indicate abnormal compensatory HR acceleration in late exercise. Healthy individuals exhibit zero or negative ΔHR–WR slopes (no change or deceleration of HR response [82]). In patients with advanced CAD who cannot increase their HR (a condition present also in patients with autonomic dysfunction or chronotropic incompetence or due to HR-limiting medications), there is an abrupt plateau or decline in SV, leading to a reduction in CO [82]. This decrease is reflected in the characteristic flattened ΔVO_2_/ΔWR [83,84]. CPET offers valuable prognostic insights into long-term mortality in patients with established CAD. For women with CAD, each 1 mL/kg/min increase in peak VO_2_ is associated with a 10% reduction in mortality [85]. Moreover, a peak VO_2_ < 16 mL/kg/min post-myocardial infarction or post-percutaneous coronary intervention indicates an increased risk of adverse events over 2 years [82]. On the contrary, even modest improvements in peak VO_2_ correlate with enhanced survival, reduced cardiovascular risk, and better quality of life [86].

### 6.6. CPET-ESE in Pulmonary Diseases

CPET-ESE provides detailed submaximal and maximal exercise data that help identify abnormal hemodynamic responses associated with PH and RV dysfunction. By analysing pulsatile pulmonary vascular pressure–flow relationships, CPET allows for assessing RV hemodynamic load, which is crucial in understanding the disease’s impact on cardiac function [87].

During the examination, various indicators can signal RV issues, such as the flattening of the interventricular septum, which leads to a D-shaped LV, suggesting RV pressure or volume overload. Additionally, impaired RV- PA coupling, assessed through the TAPSE/sPAP ratio, worsening tricuspid regurgitation (TR), and increased B-lines all point to elevated LV filling pressures and increased ventricular interdependence [43].

Evidence demonstrates that CPET-ESE can unmask different RV contractile reserve phenotypes in heart failure patients. Importantly, RV dysfunction at rest does not necessarily indicate a poor adaptive response during exercise. By integrating CPET-derived gas exchange parameters with echocardiographic markers such as TAPSE and PASP during exercise, it is possible to non-invasively assess RV-to-pulmonary circulation coupling and contractile reserve. This has important clinical implications, as impaired RV reserve is associated with reduced ventilatory efficiency, increased ventilatory oscillations, and a higher risk of adverse events. Furthermore, exercise-induced changes in PASP and the presence of significant mitral regurgitation at rest are strong correlates of impaired RV performance. Therefore, CPET-ESE provides a powerful tool to identify RV dysfunction that is not evident at rest and may help stratify risk and guide advanced therapeutic decisions [88].

Additionally, CPET is particularly useful in differentiating the exertional limitations caused by chronic obstructive pulmonary disease or interstitial lung disease from those primarily driven by PH [89]. Blanco et al. showed that PH significantly reduces peak VO_2_ and oxygen pulse volume, indicating an interaction between ventilatory limitation and cardiovascular restriction [90]. Reduced peak VO_2_ is a strong prognostic marker for disease progression and mortality in PH patients [26,87], as well as RV strain and TAPSE/sPAP [91]. Lower TAPSE/PASP values correlate with higher disease severity and independently predict overall survival and disease severity in pulmonary arterial hypertension, performing better than other echocardiographic indices of RV function by integrating both RV contractility and pulmonary afterload [92]. Also, the mPAP/CO slope is valuable for characterising pulmonary circulation during exercise. Unlike the absolute values of mPAP, the mPAP/CO slope remains largely unaffected by variations in workload, providing a more stable and reliable measure. It is particularly relevant in pulmonary diseases, as an increased slope is associated with worse survival across various cardiopulmonary conditions [93].

Another application of CPET-ESE is the assessment of patients following an episode of pulmonary embolism (PE). CPET-ESE can provide objective insights into exercise limitation patterns, distinguishing between ventilatory inefficiency and insufficient cardiocirculatory reserve, essential for diagnosing the sequelae of PE, such as chronic thromboembolic pulmonary hypertension (CTEPH) and post-PE impairment. At three months post-PE, approximately 50% of patients exhibit some degree of cardiopulmonary exercise limitation, with ventilatory inefficiency being the predominant abnormality. This pattern persists in nearly 45% of patients at twelve months, highlighting the potential long-term impact of PE on pulmonary and cardiovascular function [26]. CPET effectively identifies these abnormalities by evaluating parameters such as VE/VCO_2_ and peak oxygen pulse. In patients diagnosed with CTEPH, severe ventilatory inefficiency is a consistent finding, but insufficient cardiocirculatory reserve is also commonly present. These findings underscore CPET diagnostic utility in differentiating post-PE impairment from deconditioning, particularly in cases where echocardiographic findings at rest are inconclusive [94]. Among patients without significant chronic comorbidities, those who experienced an intermediate- or high-risk PE event are significantly more likely to exhibit persistent or worsening cardiopulmonary limitation. This highlights the importance of CPET in the early identification of at-risk individuals, guiding further investigations such as ventilation–perfusion lung scans and, when indicated, right heart catheterisation. Current recommendations emphasise structured follow-up after PE, and CPET-ESE offers valuable insight for guiding risk stratification, functional recovery, and individualised rehabilitation strategies. While not yet a standard component of routine post-PE evaluation, its ability to provide objective and reproducible measurements of functional impairment suggests that it could play a greater role in the comprehensive management of PE survivors [26].

## 7. Conclusions and Clinical Perspectives

CPET-ESE has emerged as a crucial diagnostic and prognostic tool in assessing cardiovascular and pulmonary diseases. Its combined approach provides a comprehensive understanding of functional capacity, helping clinicians evaluate the cardiovascular system and the pulmonary and peripheral responses to exercise. Beyond diagnosis, CPET-ESE is essential for risk stratification and prognosis. It allows clinicians to identify high-risk patients who may benefit from early intervention, individualised treatment strategies, or closer follow-up. The prognostic value of CPET parameters such as peak VO_2_ and VE/VCO_2_ slope is well-established across various cardiovascular conditions, helping to predict adverse events, disease progression, and mortality [70]. Applications of CPET-ESE are increasing, as traditionally, this method was reserved for evaluating candidates for heart transplantation or mechanical support. However, recent European guidelines have expanded their use to assess exercise intolerance severity and evaluate BP responses to exercise [7,95]. Importantly, CPET-ESE is not limited to patients with overt cardiovascular disease. It is also valuable in identifying early functional impairment in asymptomatic individuals and guiding exercise-based rehabilitation programmes. The CPET-ESE protocol is not readily available in all clinical settings due to several technical and logistical limitations, including high costs, time requirements, the need for equipment and trained personnel, and the possibility that some patients may be unable to perform physical exertion. Nonetheless, integrating CPET-ESE into routine clinical practice can enhance patient outcomes by improving diagnostic accuracy, refining therapeutic decisions, and optimising risk management. Despite its clinical potential, the broader implementation of CPET-ESE is limited by heterogeneity in protocols and the need for advanced operator training.

Hence, as research advances, future developments in CPET-ESE may further expand its clinical applications, focusing on a more individualised approach to diagnosis and treatment, leading to improved patient care. Multicenter prospective studies are required to standardise CPET-ESE protocols and define clear indications. Moreover, research should focus on cost-effectiveness, training requirements, and integration into clinical workflows. By bridging the gap between cardiac and pulmonary physiology, CPET-ESE stands as a cornerstone of modern cardiopulmonary evaluation, offering a more precise and personalised approach to cardiovascular care and allowing more effective management strategies.

This review highlights the rationale, protocol, and clinical relevance of CPET-ESE and encourages the broader implementation of this powerful diagnostic strategy to fill current gaps in the evaluation of exertional symptoms. By synthesising current insights and identifying gaps in knowledge, this narrative review underscores the evolving role of integrated CPET-ESE protocols in the non-invasive assessment of functional and hemodynamic limitations—a promising field for further clinical validation and innovation.

## Figures and Tables

**Table 1:** Cardiopulmonary exercise testing: parameters and interpretation.

Parameters	Significance	Normal Values	Interpretation
*Exercise Intolerance*			
**Heart rate (HR) Response**	An increase in HR during exercise is a sign of healthy cardiovascular function. HR reserve is calculated as the difference between the age-predicted maximal HR and resting HR. The chronotropic response is calculated as 100∙(peak HR—resting HR)/HR reserve [20].	HR should increase continuously and exceed 85% of the age-predicted maximal HR at peak exercise, indicating maximal effort.	The inability to achieve ≥80% of the HR reserve, or >62% in patients on beta-blockers, is termed chronotropic incompetence. This abnormality can be observed in several pathologic conditions, e.g., HF, CAD, sick sinus syndrome, and atrioventricular block [20].
**Blood pressure (BP) Response**	Variations in BP during exercise result from an integrated response by the autonomic nervous and cardiovascular systems.	SBP increases proportionally with workload (with a steeper SBP/workload slope in women), whereas DBP remains relatively stable or decreases slightly due to vasodilation in active muscles [21].	An abnormal BP response (both hypotensive and hypertensive) contributes to reduced functional capacity. It usually indicates impaired cardiovascular adaptation and/or autonomic dysfunction and has poor prognostic implications in patients with cardiovascular disease (e.g., ischemic cardiomyopathy and severe aortic stenosis) [21,22].
**Oxygen consumption (VO_2_)**	Maximal oxygen uptake during exercise typically averaged over 30 s [23]. It is an indicator of global cardiopulmonary and skeletal muscle function.	An absolute peak VO_2_ > 20 mL/kg/min and/or >80% of the predicted value (based on age, sex, weight, and height as per the Wassermann–Hansen equation [24]) indicates good functional capacity and favourable prognosis [25] (predicted values might be less accurate in HFpEF [2]).	Values < 16 mL/kg/min suggest a poor prognosis in HF [15]. Values ≤ 14 mL/kg/min, or ≤12 mL/kg/min in patients on beta-blockers, represent an indication for heart transplant in patients with advanced HF.
**VO_2_/HR (oxygen pulse)**	The amount of oxygen consumed per heartbeat. It represents an indirect measure of SV during exercise based on Fick’s principle (VO_2_ = HR∙SV∙AVO_2_diff), assuming a constant AVO_2_diff.	This increases steadily throughout the exercise and may plateau near the end of the test. It should reach at least 80% of the predicted level.	An impaired oxygen pulse, with or without a prolonged plateau, often signals impaired SV and/or CO, a common feature in HF.
**Respiratory exchange ratio (RER)**	VCO_2_/VO_2_ ratio, reflecting exercise intensity.	Values ≥ 1.00 signal that the patient has reached the anaerobic threshold.	Used to confirm that maximal effort has been achieved. Psychogenic hyperventilation may lead to falsely elevated values, particularly during the initial minutes of exercise.
		Values ≥ 1.10 signal maximal effort.	
**VO_2_/work slope**	Used to assess exercise limitation due to physical deconditioning or LV dysfunction.	A normal VO_2_/work slope is about 10 mL/min/W and remains stable regardless of exercise duration.	A flattening (i.e., plateauing) or downsloping VO_2_/work slope is associated with reduced CO, often due to myocardial ischemia, severe aortic stenosis, or advanced HF.
*Ventilatory Efficiency*			
**Minute ventilation to carbon dioxide production (VE/VCO_2_) slope**	Reflects ventilation–perfusion matching in the lungs [16].	A VE/VCO_2_ slope < 30 indicates normal ventilation–perfusion matching during exercise. Values > 36 indicate significant ventilation–perfusion mismatch.	Substantial prognostic value in HF or pulmonary disease, even during submaximal exercise [16].
	It is influenced by CO_2_ production during exercise, the VD/VT, and PaCO_2_.		
**End-tidal CO_2_ pressure (PETCO_2_)**	Reflects alveolar ventilation and perfusion matching. It is a reliable, non-invasive estimate of PaCO_2_	36–42 mmHg at rest. A slight increase (3–8 mmHg) is observed during the exercise before reaching the AT, usually followed by a decline at peak exercise.	A further increase after the AT may indicate hypoventilation or poor ventilatory efficiency [12].
**Breathing reserve (BR)**	The difference between MVV (measured directly or estimated based on FEV_1_) and VE. It indicates the ventilatory response to exercise.	>11 L/min or ≥30% when adjusted for MVV.	A reduced BR, where VE is close to MVV, is frequently observed in primary lung diseases, particularly chronic obstructive lung conditions [17].
**Dead space to tidal volume (VD/VT) ratio**	Represents the ratio between physiological dead space (VD) and tidal volume (VT). It reflects pulmonary ventilation–perfusion interactions. It decreases during exercise due to alveolar recruitment and pulmonary vasodilation.	At rest: 33–34%. It should decrease during exercise, with normal peak VD/VT values ≤ 25%.	Increased peak VD/VT may indicate pulmonary dysfunction and ventilation–perfusion mismatch due to pulmonary hypertension, left-sided pulmonary hypertension, and impaired gas exchange [19,26].
**Exercise Oscillatory Ventilation (EOV)**	Cyclic ventilation and gas exchange fluctuation during exercise. To diagnose EOV, rhythmic fluctuations must occur during at least 60% of the exercise, with an amplitude > 15% above the average resting value [27].	Absent.	It indicates poor prognosis, especially in patients with advanced HF.
*Peripheral response to exercise*			
**Arterial-venous oxygen difference (AVO_2_diff)**	Indicates oxygen extraction by muscles during exercise. It can be indirectly estimated using Fick’s principle, with CO measured via invasive catheterisation or ESE.	At rest: about 5 mL/100 mL; it can rise to nearly 16 mL/100 mL at peak exercise in healthy individuals [14].	Low values indicate impaired muscle oxygen extraction or circulatory abnormalities. Anaemia must be ruled out as a potential contributing factor to impaired oxygen delivery and AVO_2_diff [12].

AT, anaerobic threshold; CAD, coronary artery disease; CO, cardiac output; CPET, cardiopulmonary exercise testing: CV, cardiovascular; DBP, diastolic blood pressure; ESE, exercise stress echocardiography; HF, heart failure; LV, left ventricle; MVV, maximal voluntary ventilation; PaCO_2_, arterial CO_2_ partial pressure; RV, right ventricle; SBP, systolic blood pressure.

**Table 2:** Exercise stress echocardiography: parameters and interpretation.

Parameters	Significance	Normal Values	Interpretation
*Left Ventricle*			
**Left ventricle ejection fraction (LVEF)**	Key parameter used to classify patients with HF and prognostic indicator when below 50% [28]. It cannot provide direct information about myocardial contractility, as it is influenced by load, heart rate, and geometry [12].	62 ± 5% at rest, slightly higher in women than in men [11].	Values < 30% at rest or during exercise are associated with poor prognosis [28]. An increase of at least 7.5% during exercise indicates adequate contractile reserve. An increase <7.5% suggests restricted coronary flow reserve and/or myocardial damage, even with normal resting LVEF.
**Wall Motion Score Index (WMSI)**	It describes the presence of regional wall motion abnormalities [29].	A score of 1 indicates normal regional wall motion in all the segments that are being considered (usually 16 or 17).	Values > 1 indicate regional wall motion abnormalities (e.g., hypokinesia, akinesia, or dyskinesia), as seen in ischemia, infarction, myocarditis, Takotsubo cardiomyopathy, etc. Values ≥ 1.3–1.5 are associated with a worse prognosis [30].
**Left ventricle stroke volume (LV SV)**	Represents the volume of blood ejected by the LV with every heartbeat. It is estimated by multiplying the LV outflow tract area by the velocity–time integral of the LV outflow tract, measured using pulsed-wave Doppler [29].	Normal values are 60–100 mL/beat in healthy adults at rest. BSA-indexed normal values are ≥40 mL/m^2^ for men and ≥ 32 mL/m^2^ for women [31]. An exercise-induced increase in LV SV of 20% or more [29] is termed flow reserve.	Inadequate flow reserve has been shown to predict the risk of pulmonary oedema in different cardiovascular conditions [32]. Furthermore, it is associated with worse outcomes in patients with HF [33]. In patients with AS, it correlates with adverse LV remodelling and increased myocardial stress, representing an early marker of LV decompensation [34].
**Tissue Doppler imaging-derived systolic tissue velocity (TDI-S’)**	Early systolic velocity of the mitral annulus. It can be reported as individual values for specific LV segments (septum or lateral wall) or as an average.	It correlates with peak VO_2_, making it useful in exercise testing, but no universally accepted threshold defines an abnormal tissue TDI-S’ at peak effort.	It has been proposed as a more reliable indicator of LV contractility than LVEF for diagnostic and prognostic uses [35].
**Mitral E/e’**	E-wave is the early diastolic transmitral flow velocity measured by pulsed-wave Doppler; e’ is the early diastolic velocity of the mitral annulus (septum or lateral wall), measured by TDI [36].	It is commonly used to estimate LV filling pressures. However, several studies raised doubts about the reliability of this parameter as a non-invasive estimator of LV filling pressure, particularly during exercise [37]. Normal diastolic response to exercise includes an E/e′ ratio ≤ 14, septal e′ velocity > 7 cm/s and TRV < 3.1 m/s [29].	The E/e′ ratio is a prognostic marker in patients with HF and other cardiac conditions [36]. An increased E/e′ ratio (>14) usually indicates elevated LV filling pressures [36].
**Left ventricle global longitudinal strain (LV GLS)**	It measures LV myocardial contractility using speckle tracking [11]. It represents the change in LV myocardial length from end-diastole to end-systole, normalised to end-diastolic length (conventionally indicated with a negative value) and averaged across the three apical views.	<−20%, but highly vendor-dependent [38].	During exertion, changes in GLS provide clinically relevant information in conditions such as HF, hypertrophic cardiomyopathy (HCM), and heart valve disease [29]. In particular, a blunted increase in GLS during exertion (<2%) indicates limited contractile reserve [29].
*Left atrium*			
**Left atrial reservoir function (LARS)**	It measures LA function during the reservoir phase (i.e., LV systole) using speckle tracking [39].	>39% in healthy individuals at rest. The LARS/E/e’ ratio has been shown to correlate with LV filling pressures, improving discrimination between HF and non-cardiac dyspnoea [39].	Values < 23% at rest are considered abnormal and are associated with adverse LA remodelling, increased risk of atrial fibrillation [40], elevated LV filling pressures, RV-PA uncoupling [39], and impaired ventilatory efficiency during exercise [41]. It has prognostic significance in both HFrEF and HFpEF [42]. Exercise-induced reduction in LARS suggests worsening atrioventricular uncoupling and pulmonary hypertension [39].
*Right Ventricle and Pulmonary Circulation*			
**Systolic pulmonary arterial pressure (sPAP)**	Evaluates pulmonary hemodynamics and is estimated via echocardiography using the formula	The upper normal value is <35 mmHg at rest and <43 mmHg at exercise [29].	Values > 50–60 mmHg during exercise are associated with exercise-induced PH and represent markers of adverse outcomes [29,43]. sPAP is significantly affected by RV function and CO.
	*sPAP = 4 × (TRV)^2^ + RAP*,		
	where RAP is estimated from IVC diameter and its inspiratory collapse [44].		
**Tricuspid annular plane systolic excursion (TAPSE)**	Assesses RV longitudinal systolic function, which is significantly correlated with RV global function.	>17 mm at rest [11].	Values < 17 mm are associated with RV systolic dysfunction [11].
**TAPSE/sPAP ratio**	It expresses RV-PA coupling, which reflects the relationship between RV function and pulmonary vascular load [45].	No agreed-upon reference values. Healthy subjects have higher values than patients with cardiopulmonary diseases [43]. TAPSE/sPAP typically reduces during exercise [46].	TAPSE/sPAP < 0.70 mm/mmHg at rest or <0.5 mm/mmHg at peak exercise have been associated with poor survival and cardiac events in cardiovascular and non-cardiac conditions [46]. It is inversely related to peak VO_2_ and VE/VCO_2_ slope at peak exercise [45].
**Mean pulmonary artery pressure to cardiac output (mPAP/CO) slope**	It reflects the increase in mPAP during exercise, normalised for the rise in CO [9,47,48].	A value < 3 mm Hg·L^−1^·min^−1^ indicates a physiological pulmonary hemodynamic response to exercise [9]. Echocardiography can only estimate mPAP indirectly [49].	A steeper slope has been associated with poor survival and cardiac events in different cardiovascular and extracardiac conditions [9,46,47,48].
*Heart valve disease*			
**Vena contracta**	It is the narrowest region of a regurgitant jet just downstream from the regurgitant orifice. Measuring the vena contracta width via colour Doppler echocardiography provides a semi-quantitative assessment of regurgitation severity [50]. Technical factors and flow conditions can influence measurements.	<0.3 cm suggests mild regurgitation.	A wider vena contracta correlates with more severe regurgitation for AR and MR. If valve insufficiency worsens with exertion, values may increase.
		≥0.7 cm suggests severe regurgitation.	
**Effective Regurgitant Orifice Area (EROA)**	It quantifies the size of the effective opening through which blood regurgitates, providing a quantitative measure of regurgitation severity. It is commonly calculated using the PISA method [50]. Technical factors and flow conditions can influence measurements.	<0.20 cm^2^ indicates mild regurgitation [50].	Higher EROA values indicate more significant regurgitation (≥0.40 cm^2^ for severe MR and TR; ≥0.30 cm^2^ for severe AR). The thresholds may be lower in secondary/functional regurgitation due to differences in pathophysiology and clinical implications [50]. If valve insufficiency worsens with exertion, values may increase.
**Peak velocity**	Doppler-derived measurements that assess the severity of heart valve stenosis by evaluating the velocity of blood flow across the valve [51].	Peak aortic jet velocity: <2.5 m/s.	Aortic stenosis [51]: 2.5–3.0 m/s (mild stenosis); 3.0–4.0 m/s (moderate stenosis); >4.0 m/s (severe stenosis).
**Mean pressure gradient (MPG)**	Doppler-derived measurements that assess the severity of heart valve stenosis by evaluating gradient across the valve [51]. Measurements are flow-dependent and can be influenced by factors such as CO, BP, and arterial compliance.	Aortic MPG: <10 mmHg; Mitral MPG: <2.5 mmHg.	Aortic stenosis MPG [29,51]:
			<20 mmHg (mild stenosis);
			20–40 mmHg (moderate stenosis);
			>40 mmHg (severe stenosis);
			Δ(peak–rest) > 20 mmHg (severe stenosis) [18].
			Mitral stenosis MPG [29,51]:
			<5 mmHg (mild stenosis);
			5–10 mmHg (moderate stenosis);
			>10 mmHg (severe stenosis);
			>15 mmHg during exercise (severe stenosis) [18].
*Congestion assessment*			
**B-lines**	Lung ultrasound can quantify EVLW at rest and during exercise [13].	Absence of B-lines at rest and minimal increase during exercise.	

AR, aortic regurgitation; AS, aortic stenosis; BSA, body surface area; E, early diastolic transmitral flow velocity; E/e’ ratio, ratio of early diastolic mitral inflow velocity to early diastolic mitral annulus velocity; ESE, exercise stress echocardiography; EVLW, extravascular lung water; HCM, hypertrophic cardiomyopathy; HF, heart failure; IVC, inferior vena cava; LV, left ventricle; MR, mitral regurgitation; PA, pulmonary artery; PH, pulmonary hypertension; PISA, Proximal Isovelocity Surface Area; RAP, right atrial pressure; RV, right ventricle; STE, speckle tracking echocardiography; TRV, tricuspid regurgitation velocity; TRV, tricuspid regurgitation velocity; VCO_2_, carbon dioxide production; VE, minute ventilation; VO_2_, oxygen consumption.

## Data Availability

Data sharing is not applicable. No new data were created or analysed in this study.
